# Impact of salinomycin on the intestinal microflora of broiler chickens

**DOI:** 10.1186/1751-0147-49-30

**Published:** 2007-10-26

**Authors:** Charlotte H Johansen, Lotte Bjerrum, Karl Pedersen

**Affiliations:** 1 National Veterinary Institute, Technical University of Denmark, Hangøvej 2, DK-8200 Aarhus N, Denmark; 2 Department of Veterinary Pathobiology, Faculty of Life Sciences, University of Copenhagen, Stigbøjlen 7, 1870 Frederiksberg C, Denmark

## Abstract

**Background:**

The ionophoric coccidiostat salinomycin is widely used in chicken feed. In the near future the use of ionophore coccidiostats may be banned as has been the case for other antimicrobial growth promoters. This study was conducted to examine the effect of salinomycin on *Campylobacter jejuni *infection and on the composition of the caecal microflora in broiler chickens.

**Methods:**

An experimental infection study was carried out in isolators and the intestinal microflora was analyzed using quantitative cultivation, denaturant gradient gel electrophoresis (DGGE), cloning and sequencing.

**Results:**

We found no effect of salinomycin on *C. jejuni *but salinomycin significantly affected the composition of the microflora. In addition, salinomycin significantly reduced the prevalence of *Clostridium perfringens *and we observed a significant increase (62%) in the mean body weight of salinomycin treated chickens compared to un-treated controls.

**Conclusion:**

Termination of the use of ionophore coccidiostats will not affect food safety related to campylobacter, but will increase the risk of necrotic enteritis in the broilers.

## Background

Salinomycin is an ionophoric coccidiostat, which is widely used as a supplement in poultry feed to control infection with coccidia [1]. In addition, it is known that salinomycin, has an inhibitory effect on *Clostridium perfringens*. Thus, the use of salinomycin leads to a decrease in the incidence of necrotic enteritis in broiler chickens [2-7]. It has also been reported that salinomycin can reduce the prevalence of salmonella in chickens [1]. However, Scalzo et al. [8] found no reduction in the frequency or the level of salmonella shedding by the use of salinomycin. In fact, they observed an increase of one log unit on salmonella colony forming units (cfu) between control and salinomycin treated chickens.

By 2006, the use of all antimicrobial growth promoters was prohibited in the European Union. Also the use of ionophore coccidiostats is expected to be banned in the near future [6]. The consequences of such a ban on the incidences of *C. perfringens *related diseases or the prevalence of salmonella and campylobacter is essentially unknown. Likewise, the action and selective pressure of salinomycin on the normal intestinal microflora of broiler chickens is still not clearly elucidated.

The purpose of this study was to examine the effect of salinomycin on *Campylobacter jejuni *infection and on the composition of the microflora of the caecum in broiler chickens. This was completed using experimental infection and bacterial culturing. Additionally, the DNA fingerprinting technique Denaturant Gradient Gel Electrophoresis (DGGE) and cloning and sequencing of 16s rDNA was carried out.

## Methods

### Animals, experimental design, sampling and cultivation

The chickens used in this study were conventional broiler chickens (Ross) of mixed sex, purchased as day-old from a local hatchery (DanHatch A/S, Randers, Denmark). The chickens were transferred directly from the hatchery to the experimental unit, where they were housed in isolators (Montair Andersen B.V. HM 1500, The Netherlands).

Initially, transport-boxes, feed and water samples from each isolator, and cloacal swabs from 5 randomly selected chickens from each isolator, were analyzed for the presence of campylobacter by cultivation.

The chickens were divided into 6 experimental groups (Table 1). The 6 groups, each with an initial size of 37 day-old chickens, were kept in separate isolators. The chickens had access to feed and water *ad libitum*. They were fed a conventional wheat based broiler feed without antimicrobial additives (Faculty of Agricultural Sciences, University of Aarhus, Denmark). However, the diet of group 1 and 2 was supplemented with salinomycin (75 mg/kg) from day 8 and onwards. At day 14, all chickens from group 1 to 4, were orally inoculated with approximately 1 × 10^9 ^cfu of *C. jejuni *strain DVI-sc181. The control groups (group 5 and 6) were given a 0.9% NaCl solution. The inoculation was carried out inside the isolators by individual oral gavage of 500 μl of a bacterial suspension or a NaCl solution, using a 1 ml syringe with an attached flexible tube.

**Table 1:** Study design

Isolator/Group	Salinomycin from day 8	C. jejuni at day 14
1	+	+
2	+	+
3	-	+
4	-	+
5	-	-
6	-	-

Four groups (1–4) of 14-day old broiler chickens (37 birds per group) were infected orally with *Campylobacter jejuni *(10^9 ^colony forming units). Groups 1 and 2 were given feed containing salinomycin from day 8. Two additional groups (5 – 6) were kept as controls.

Samples were taken at day 7, 13, 16, 23, 30 and 36, respectively. At each sampling 6 birds were removed from each group and killed by decapitation. The gastrointestinal tract was excised and the contents of the ileum and caecum were collected. The intestinal contents from 3 chickens were pooled by segment before further analysis. Quantitative cultivation of *C. jejuni, C. perfringens *and lactobacilli was carried out from caecal and ileal contents of the chickens at each sampling. The samples were diluted in 10-fold steps in buffered peptone water (BPW: Merck 1.07228) and quantification was done by the spread-plate method on mCCDA (Oxoid, Basingstoke, UK), incubated microaerophilically at 42°C for 48 h. Likewise, lactobacilli were enumerated on Rogosa agar (Merck 5413) after anaerobic incubation at 37°C for 48 h. *C. perfringens *were counted as black colonies surrounded by a precipitation zone on Tryptose-sulphite-cycloserine agar [9] containing egg-yolk after anaerobic incubation at 37°C for 24 h. Furthermore, samples from the caecum and ileum were collected in Eppendorf tubes, for a 16S rDNA-based study of the microbial community, and stored in a 3 times volumen of ethanol at 4°C until further processing. The experiment was carried out in accordance with the guidelines from the Danish Ministry of Justice with respect to animal experiments.

### DNA extraction

DNA was extracted from the caecal contents. An amount of 1.6 ml of caecal material suspended in ethanol was centrifuged at 10,000 rpm for 1 min. The supernatant was discarded and the sample was washed with BPW and centrifuged at 10,000 rpm for 1 min. The washing step was repeated. Finally, the DNA was extracted using the QIAamp DNA Stool Mini Kit (Qiagen Inc., Hilden, Germany). The extraction was carried out in accordance with the instructions of the manufacturer, with an additional step of lysozyme treatment, which was added to the procedure before the use of InhibitEX tablets. An amount of 140 μl of a 10 mg/ml solution of lysosyme (Sigma-Aldrich, St. Louis, MO, USA) in Tris-EDTA buffer (10:1 mM), pH 8, was added to each extraction tube and the samples were incubated at 37°C for 30 min. The DNA was eluted in 200 μl buffer AE (Qiagen) and stabilised by adding 4 μl of a 40 mg/ml bovine serum albumin (Ambion, Cambridgeshire, UK) and 2 μl of Ribonuclease-A. All DNA samples were stored at -20°C until further processing.

### PCR amplification with HDA (universal 16S rDNA)primers

PCR amplifications of total bacterial community DNA were performed using the primers HDA1-GC (5'-**CGC CCG GGG CGC GCC CCG GGC GGG GCG GGG GCA CGG GGG G**AC TCC TAC GGG AGG CAG CAG T-'3 ; GC-clamp in boldface) and HDA2 (5'-GTA TTA CCG CGG CTG CTG GCA C-3') (DNA-Technology, Aarhus, Denmark). The thermocycling programme was: 94°C for 4/12 min; 25 cycles of 94°C for 30 s, 56°C for 30 s, and 68°C for 1 min; and finally 68°C for 7 min [10]. PCR was performed in 0.2 ml tubes with a Peltier Thermal Cycler (PTC) 200 (MJ Research Inc, Watertown, MA, USA) and a reaction mixture as previously described [11]. The PCR products were confirmed by electrophoresis on a 2% agarose gel containing 0.1 μg/ml ethidium bromide (Bio-Rad, Hercules, CA, USA) and viewed by UV transillumination.

### DGGE analysis

DGGE was performed with the Dcode universal mutation detection system (Bio-Rad) using 16 cm by 16 cm by 1 mm gels. The 8% polyacrylamide gels (ratio of acrylamide:bisacrylamide, 37.5:1) (Bio-Rad) contained a 30 to 55% gradient of urea and formamide (Fluka, Sigma-Aldrich, St. Louis, MO, USA) increasing in the direction of electrophoresis, which was run at 130 V and 60°C for 4 h. The gels were stained with SybrGold (1:10,000 dilution) (Bie & Berntsen, Herlev, Denmark) and viewed by UV transillumination [11].

The DGGE ladder used in this experiment was prepared from individual pure cultures (*Lactobacillus johnsonii, Lactobacillus acidophilus, C. jejuni, Escherichia coli *and *C. perfringens*) as previously described [11].

The intestinal bacterial community profiles were compared using the BioNumerics software (Applied Maths BVBA, Belgium). Initially, the DGGE gels were normalized by means of the DGGE markers used, and the software conducted a band search according to a 5% minimum profiling and a 10% grey-zone interval. Subsequently, all bands were checked manually. The comparisons were based on the Dice similarity coefficient and the un-weighted pair group method using arithmetic averages (UPGMA) for clustering.

### Cloning and sequencing

DNA samples from the caecum of three 7-day-old chickens and three caecum samples from the 30-day-old chickens were cloned using primer pair 26F (5'-AGA GTT TGA TCC TGG CTC A-3') and 1390R (5'-GAC GGG CGG TGT GTA CAA-3') (DNA Technology) and the following amplification programme: 1 min at 93°C followed by 25 cycles of 30 s at 92°C, 60 s at 57°C, and 45 s at 72°C. In the last cycle, the 72°C step was extended for 5 min, and the samples were finally cooled down to 4°C. The PCR product was purified using the QIAquick PCR purification kit (Qiagen) and cloned into *E. coli *using the Topo XL cloning kit (Invitrogen, Carlsbad, CA, USA) as specified by the manufacturer. All clones were checked by DGGE (using primer pair HDA1-GC and HDA2) and selected for sequencing on the basis of their migration in the gel. In total 100 clones (30 from the 7-day-old chickens and 70 from the 30-day old chickens) were screened. Plasmid DNA of the selected clones was purified using the GenElute Plasmid Miniprep Kit (Sigma-Aldrich). For sequencing of clones the Dyenamic ET Terminator Cycle Sequencing Kit from Amersham Biosciences and primer 341F (5'-CCC ACG GGA GGC AGC AG-3') (DNA Technology) were used and the sequencing was carried out on an ABI 3100 capillary DNA analysing system. The retrieved sequences were compared with the Genbank database using BLAST algorithm [12].

### Phylogenetic analysis

The clones were analyzed in order to visualize their similarities to known bacterial species. Multiple sequence alignments of the nucleotide sequences were performed using the CLUSTAL W program [13] and the alignment editor GENEDOC [14]. The phylogenetic analysis was made using the software PHYLIP [15] and TREEVIEW [16]. A neighbour joining tree was generated on the basis of 280 base pair sequences and the robustness of the tree was evaluated by the bootstrapping-resembling method with 1000 replicates.

## Results

### Bacterial counts

No campylobacter was detected in the transport boxes, the feed and water samples or in the day-old chickens.

The results of the bacterial counts are shown in Tables 2 (ileum) and 3 (caecum). A persistent infection with *C. jejuni *was established in both the ileum and the caecum of the chickens from the day of infection and throughout the experiment. The counts of *C. jejuni *were 1 to 2 orders of magnitude higher in the caecal samples compared to the ileal samples. There was no significant difference in *C. jejuni *counts in infected birds treated with salinomycin (groups 1 and 2) compared to infected but untreated groups (3 and 4), with one exception at day 30, where the *C. jejuni *counts were significantly higher in the salinomycin treated chickens, in both the ileum and the caecum.

**Table 2:** Bacterial counts (log10 cfu/g) from the ileal content of the chickens

		*Campylobacter*						*Clostridium perfringens*						Lactobacilli					
		
Time (day)		7	13	16	23	30	36	7	13	16	23	30	36	7	13	16	23	30	36
				
Group	Treatment																		
																		
1 – 1	Salinomycin +	nd	nd	5.04	6.94	7.41	6.04	nd	3.23	nd	nd	nd	nd	<10^5^	4.30	4.97	7.34	6.69	8.72
- 2	*Campylobacter*	nd	nd	5.45	6.95	6.86	6.23	nd	3.28	nd	nd	nd	nd	<10^5^	4.26	5.71	8.18	6.62	8.63
2 - 1	Salinomycin +	nd	nd	6.18	7.52	7.80	6.04	4.69	ne	nd	nd	nd	2.70	<10^5^	8.30	8.89	8.94	7.95	8.57
- 2	*Campylobacter*	nd	nd	5.60	6.97	6.61	7.18	6.92	nd	nd	nd	nd	2.30	<10^5^	ne	9.76	8.89	9.04	8.89
3 - 1	*Campylobacter*	nd	nd	6.73	ne	6.30	6.28	ne	2.60	3.48	4.04	3.11	3.59	<10^5^	7.51	8.26	8.71	8.15	8.90
- 2		nd	nd	4.70	6.95	6.48	5.15	6.85	2.48	4.00	3.61	3.26	6.46	<10^5^	8.62	8.52	9.15	8.67	8.48
4 - 1	*Campylobacter*	nd	nd	7.32	6.18	6.08	6.04	6.86	4.66	4.41	4.11	2.95	5.54	<10^5^	7.94	7.91	7.18	7.67	9.34
- 2		nd	nd	7.08	6.15	5.30	6.28	5.52	2.95	5.11	6.18	nd	3.94	<10^5^	8.08	8.00	7.53	7.49	9.08
5 - 1	Control	nd	nd	nd	nd	nd	nd	5.30	3.61	3.28	3.79	5.18	5.40	<10^5^	9.65	9.04	8.28	7.15	7.64
- 2		nd	nd	nd	nd	nd	nd	5.59	2.90	3.91	5.11	6.34	6.23	<10^5^	8.77	7.75	7.78	8.11	7.94
6 - 1	control	nd	nd	nd	nd	nd	nd	5.38	5.18	2.48	nd	nd	nd	<10^5^	8.76	8.99	8.28	8.23	7.61
- 2		nd	nd	nd	nd	nd	nd	6.26	2.95	2.30	nd	nd	5.04	<10^5^	8.93	8.66	8.90	8.67	6.40

Groups 1 – 4 were infected with *Campylobacter jejuni *as 14-day-old. Two of these groups (1 – 2) were given feed containing salinomycin from day 8. Groups 5 – 6 were kept as control chickens. The detection limit for bacterial counts was 10^2^. nd = none detected, ne = not examined

**Table 3:** Bacterial counts (log10 cfu/g) from the caecal content of the chickens

		*Campylobacter*						*Clostridium perfringens*						Lactobacilli					
		
Time (day)		7	13	16	23	30	36	7	13	16	23	30	36	7	13	16	23	30	36
				
Group	Treatment																		
																		
1 - 1	Salinomycin +	nd	nd	7.3	8.45	8.65	7.98	nd	ne	nd	nd	nd	nd	<10^5^	7.76	7.95	8.00	7.32	8.80
- 2	*Campylobacter*	nd	nd	6.48	7.58	9.28	7.88	ne	ne	nd	nd	3.97	nd	<10^5^	8.41	7.81	7.98	7.77	8.20
2 - 1	Salinomycin +	nd	nd	7.28	7.88	9.04	8.28	5.48	ne	nd	nd	nd	2.48	<10^5^	8.28	9.34	8.96	8.80	8.32
- 2	*Campylobacter*	nd	nd	7.08	8.41	8.40	8.04	7.40	4.48	nd	nd	nd	nd	<10^5^	8.92	9.95	9.28	9.23	9.00
3 - 1	*Campylobacter*	nd	nd	8.23	8.11	8.20	7.93	ne	4.49	5.52	3.98	3.38	3.54	<10^5^	9.90	9.26	9.18	8.99	9.79
- 2		nd	nd	7.49	8.08	7.43	7.91	ne	5.04	4.30	5.86	2.78	4.67	<10^5^	9.93	9.15	9.23	8.88	8.63
4 - 1	*Campylobacter*	nd	nd	8.41	7.81	7.56	6.42	6.48	ne	5.98	5.77	4.11	6.08	<10^5^	8.36	9.18	8.51	8.83	9.00
- 2		nd	nd	7.08	7.40	6.51	7.18	8.38	ne	4.60	4.11	3.81	5.45	<10^5^	9.64	6.86	8.15	8.86	8.98
5 - 1	Control	nd	nd	nd	nd	nd	nd	8.23	6.18	5.86	4.85	4.45	5.45	<10^5^	9.36	9.04	8.77	8.59	8.70
- 2		nd	nd	nd	nd	nd	nd	7.23	5.28	4.51	3.88	6.23	4.34	<10^5^	9.20	8.68	8.69	8.64	8.23
6 - 1	control	nd	nd	nd	nd	nd	nd	5.85	7.53	3.30	nd	nd	nd	<10^5^	9.11	9.72	8.65	8.76	8.67
- 2		nd	nd	nd	nd	nd	nd	8.15	ne	3.00	nd	nd	5.20	<10^5^	8.70	9.64	8.57	8.78	9.30

Groups 1 – 4 were infected with *Campylobacter jejuni *as 14-day-old. Two of these groups (1 – 2) were given feed containing salinomycin from day 8. Groups 5 – 6 were kept as control chickens. The detection limit for bacterial counts was 10^2^. nd = none detected, ne = not examined

In salinomycin treated chickens (groups 1 and 2),*C. perfringens *was detected in 6 out of 24 ileal samples examined and in 5 out of 20 caecal samples examined. In the remaining groups (3 to 6), which all received broiler feed without salinomycin, *C. perfringens *was detected in almost all samples, with an exception of group 6, where only 7 of 12 and 6 of 11 samples were positive in the ileum and the caecum, respectively. In the ileum, there was a significant difference between the *C. perfringens *counts in the *C. jejuni *infected, salinomycin treated chickens (groups 1 and 2) compared to the *C. jejuni *infected chickens without salinomycin treatment (groups 3 and 4) on day 16, 23, 30 and 36. In the caecum there was a significant difference between the *C. perfringens *counts in the salinomycin treated chickens compared to untreated groups on day 16, 23 and 36. With one exception (ileum, day 16) there was no significant difference in *C. perfringens *counts between *C. jejuni *infected chickens (groups 3 and 4) and the control chickens (groups 5 and 6) in neither the ileum nor the caecum of the chickens. The detection limit of our bacterial counts was 10^2 ^cfu/g.

Lactobacilli were present in all samples. *Lactobacillus *counts were slightly lower in the salinomycin treated chickens (groups 1 and 2) compared to the remaining groups (3 to 6). However, the difference was only statistically significant on day 13.

**Figure 1:**
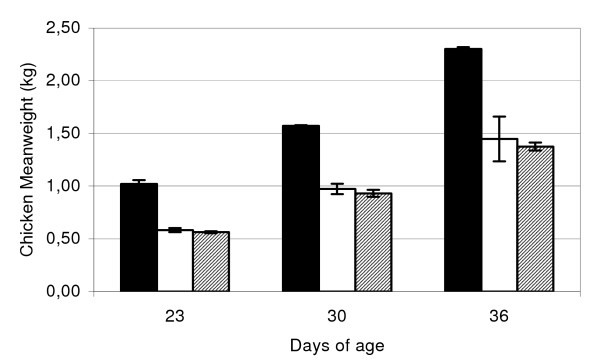
**Mean weight of the chickens at 23, 30 and 36 days of age**. Salinomycin + *Campylobacter jejuni *treated chickens (black), *C. jejuni *treated chickens (white), control chickens (hatched).

The body weight of the chickens was recorded at three samplings. The salinomycin treated chickens gained significantly more weight than non-treated chickens, at all three samplings (Figure 1). In average the groups given salinomycin in the feed had a 62% higher body weight on day 36 compared to the groups not given salinomycin. There was no significant difference in bodyweight between *C. jejuni *infected chickens (groups 3–4) and uninfected chickens (groups 5–6), which did not receive salinomycin.

### DGGE fingerprint analysis

The DGGE profiles of the caecal bacterial community of 7, 13, 16, 23 and 30-day-old chickens were analysed using BioNumerics. At day 7 the chickens did not cluster according to treatment, but samples originating from the same isolator were often more related to each other than to samples from other isolators, indicating a minor group effect. However, already from day 13 and onwards salinomycin treated chickens and control chickens clustered into two different groups, indicating an effect of salinomycin on the normal intestinal microflora. At all four sampling days, salinomycin treated chickens had a lower within-group similarity index than the control chickens (data not shown).

**Figure 2:**
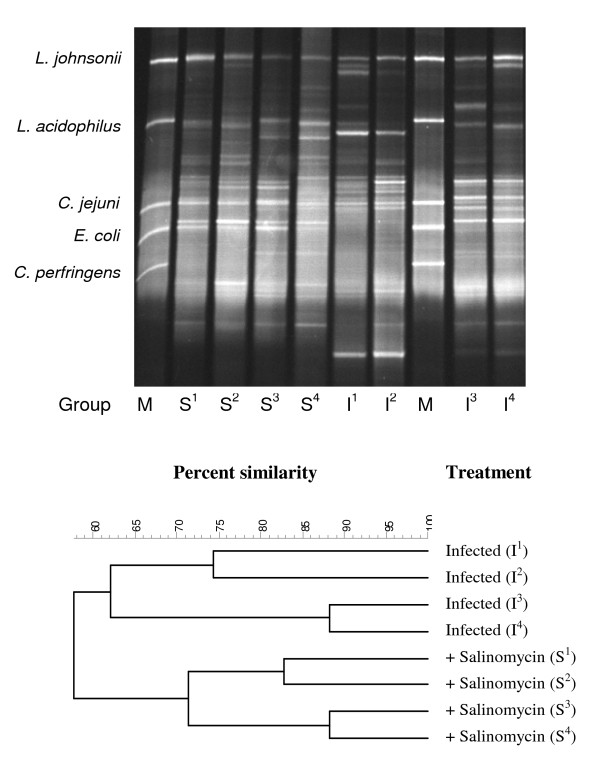
**DGGE profile and dendrogram based on caecal samples from 30-day-old chickens**. M refers to the molecular marker, containing DNA fragments from 5 pure cultures, as mentioned above. The treatment of the chickens is indicated by, I (infected with *Campylobacter jejuni*) or S (infected with *C. jejuni *+ salinomycin treated).

The DGGE band patterns of samples from day 23 and 30 (Figure 2), clustered into two distinct groups comprising the salinomycin + *C. jejuni *treated chickens (groups 1 and 2) and the *C. jejuni *infected chickens (groups 3 and 4). The similarity index between the two groups was approximately 58%. The within-group-similarity index of the salinomycin treated chickens was approximately 72% and the within-group similarity value of the non-treated, *C. jejuni *infected chickens was approximately 62%. At day 23, a 64% within-group-similarity of the salinomycin treated chickens and a 70% within-group-similarity of the non-treated, *C. jejuni *infected chickens was found. However one of the *C. jejuni *infected, non-treated samples represented the base of the dendrogram and showed only 50% similarity to the remaining samples.

### Cloning and sequencing

**Figure 3:**
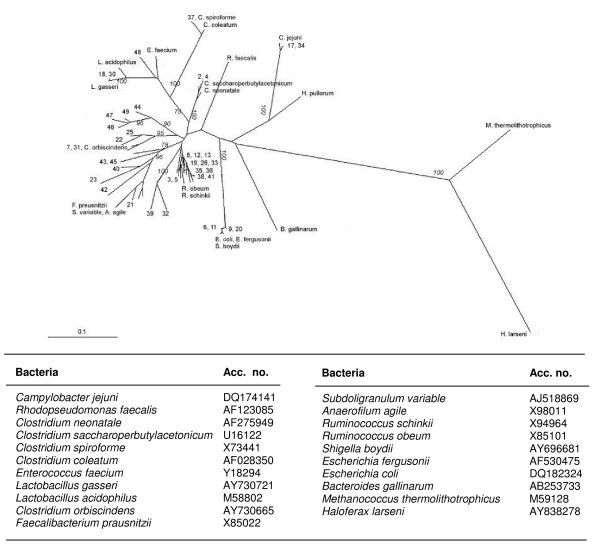
**Clustering of 40 clones (numbered) and 22 well-known bacteria (listed above), based on sequence analysis of 16s rDNA**. In the neighbor joining distance tree, branch lengths reflects genetic distances and bootstrap values, obtained from 1000 resampled datasets, are shown in *italics*.

A total of 49 clones were chosen for sequencing on basis of their different migrations in the DGGE, giving 48 useful sequences (Table 4). The caecal samples, from which the clones originated, are shown in Table 4. Blasting the sequences resulted in two clones being most closely related to *C. jejuni*, 2 to *Clostridium neonatale*, 1 to *Lactobacillus *sp. and 3 were most closely related to *Shigella boydii *and *E. coli*. Forty sequences were most closely related to an uncultured bacterium. All sequences were aligned and phylogenetically analyzed in order to identify, which groups of well-known bacteria they were related to. A neighbor joining distance tree (Figure 3) showed that 12 clones clustered with ruminococci. Seventy-five per cent of these originated from non-treated caecal samples. Another group of 7 clones clustered with *Faecalibacterium prausnitzii *and related organisms. These clones all originated from caecal samples taken at 30 days of age (3 different treatments). The remaining clones clustered among *E. coli, C. jejuni *and different clostridia or lactobacilli. Two of the clones originating from salinomycin treated chickens were related to *Clostridium *or *Lactobacillus *sp. respectively.

**Table 4:** Origin of the clones made from caecal samples

Clone no.	Age of chicken	Treatment
1–11	7 days	None
12–23	30 days	Salinomycin
24–43	30 days	*C. jejuni*
44–49	30 days	None

The age and treatment of the bird at sampling are shown.

## Discussion

In this study we found no effect of salinomycin on a *C. jejuni *infection in chickens. This is in agreement with Bolder *et al *[1] who found that salinomycin was unable to affect both the incidence and the degree of campylobacter shedding. However, by using the fingerprinting technique DGGE, we found that salinomycin had an effect on the intestinal microflora of the caecum after 5 days of treatment (Day 13) in control chickens and after 15 days of treatment (Day 23) in *C. jejuni *infected chickens. Knarreborg et al. [17] found a similar effect of antimicrobial supplementation on the composition of the microflora in the ileum of broilers. By using group specific primers these authors showed that lactobacilli and *C. perfringens *were most strongly affected by the antimicrobial treatment.

In the present study, we demonstrated no significant effect of salinomycin on the counts of lactobacilli. We found however a higher variation between lactobacilli counts in the ileum, where counts varied between 10^4 ^and 10^9 ^cfu compared to caecal counts, which varied between 10^7 ^and 10^9 ^cfu/g content. This could be explained by the fact, that the caecum is a more stable environment than the ileum. Additionally, one clone (chosen among 6 similar DGGE bands) originating from the caecum of a salinomycin treated chicken was closely related to lactobacilli, indicating conformity between the bacterial counts and the molecular analysis.

The impact of salinomycin on *C. perfringens *was very pronounced. In our study salinomycin significantly reduced the prevalence of *C. perfringens*, although *C. perfringens *was found in low numbers in some chickens. In addition, we observed a significant increase (62%) of the mean body weight in salinomycin treated chickens compared to non-treated chickens. An infection with *C. jejuni *on the other hand, did not seem to affect weight gain or body weight. This extremely high benefit from using salinomycin in the feed is difficult to explain and is more pronounced than reported for other antimicrobials [18]. Some of the important factors, associated with broiler growth depression are nutrient competition, production of metabolites or toxins and microbial deconjugation of bile salts [3,17,19]. An inhibitory effect from *C. perfringens *in untreated groups may be part of the explanation [17,20], but by analyzing our DGGE gels it is obvious, that numerous bacterial groups are involved and therefore it seems more likely that other factors play a role, too.

In this study, clones originating from salinomycin treated chickens were widely distributed among the phylogenetic groups. Thus, cloning and sequencing gave no unambiguous result concerning specific groups being affected by salinomycin. Future studies, with larger clone libraries are a necessity to elucidate this. It is noteworthy that none of the clones from 7-day-old chickens clustered with *F. prausnitzii *or lactobacilli and the phylogenetic analysis of the clones showed that the initial flora of the caecum was dominated by bacteria closely related to clostridia, ruminococci, *E. coli *and related organisms.

Analysis of DGGE fingerprints showed a lower within-group-similarity in salinomycin treated chickens compared to control chickens. This indicates, that salinomycin affects the dynamics of the gut microflora, causing the salinomycin treated chickens to be more different from each other, compared to control chickens. It also indicates that salinomycin altered the microflora, but it did not reduce the microbial diversity or number of genotypes. This is in accordance with recent observations of the effects of avilamycin, bacitracin and enramycin [18].

## Conclusion

In conclusion, salinomycin did not affect the course of a *Campylobacter *infection in broiler chickens. This observation is important and indicates that cessation of the use of ionophore coccidiostats will not affect food safety related to *Campylobacter*. However, salinomycin caused a pronounced increase in the body-weight of the chickens. Also, an effect on *C. perfringens *and on the normal intestinal microflora was observed, and the numbers of *C. perfringens *were significantly reduced by salinomycin. *C. perfringens *is normally present in the intestinal microflora of 75% to 95% of broiler chickens [21,22,22]. Thus, a stop in the use of ionophore coccidiostats will most likely lead to an increase in the cases of necrotic enteritis, to a lower performance and to an increased mortality in broiler flocks unless other measures are installed to control *C. perfringens*.

## Competing interests

The author(s) declare that they have no competing interests.

## Authors' contributions

CJH, LOB and KPE were involved in the study design and performance of the infection experiment. CJH did the DGGE work. CJH were responsible for the data analysis and manuscript preparation. KPE and LOB participated in study design and manuscript revision. All authors have read and approved the final manuscript.
